# Burden of COVID-19 on primary care in Belgium: a prospective nationwide observational study from March to August 2020

**DOI:** 10.1186/s13690-022-01003-0

**Published:** 2022-12-08

**Authors:** Bert Vaes, Bénédicte Vos, Maxime Foidart, Robrecht De Schreye, Diego Schrans, Hilde Philips, Bert Aertgeerts, Kris Doggen

**Affiliations:** 1 Department of Public Health and Primary Care, KU Leuven, Kapucijnenvoer 33, Blok J, Louvain, Belgium; 2 Department of Epidemiology and Public Health, Sciensano, Brussels, Belgium; 3 Department of Public Health and Primary Care, Ghent University, Ghent, Belgium; 4 Department of Family Medicine and Population Health, University of Antwerp, Wilrijk, Belgium

**Keywords:** COVID-19, Epidemiological monitoring, General practice, Workload, Personal protective equipment, Policy, Electronic health records

## Abstract

**Background:**

The COVID-19 outbreak had an important impact on general practice, for example the lack of personal protective equipment (PPE) and the switch to telephone consultations. We installed a monitoring instrument and reported the burden the COVID-19 pandemic placed on Belgian general practitioners (GPs) during the first wave of the pandemic.

**Methods:**

We conducted an observational longitudinal prospective study from the 19^th^ of March until the 17^th^ of August 2020. Daily data were collected by using a structured electronic form integrated into the GPs’ electronic medical records (EMRs). Data were collected on the GPs’ need for support and workload, epidemiological indicators and the availability of PPE. Reports with graphical presentations were made for GP circles and primary care zones, and governments of different administrative levels had access to all data to guide their policy.

**Results:**

A total of 3.769 different GP centres participated, which included more than 10.000 GPs. Throughout the first three weeks, 20% declared they had insufficient resources (personnel and material) for the following week. Approximately 10% continued to report this during the entire study. The majority reported being able to complete their daily tasks without loss of quality. During the first week, 30% indicated an increased workload. Afterwards, this number decreased and stabilized to an average of 10–20%. More than 70% of the consultations in March 2020 were conducted by telephone. This percentage declined in April and stabilized at approximately 30% in June 2020. Consultations due to respiratory symptoms peaked at 4000/100,000 inhabitants at the beginning of the outbreak, then decreased over time along with the COVID-19 incidence. We noticed a lack of disinfectant hand gel, surgical masks and FFP2 masks, the latter remaining problematic in the long term.

**Conclusion:**

We introduced an instrument in Belgian EMR systems to monitor the burden on GPs during the first wave of the COVID-19 pandemic. The lack of PPE and increased workload were considered to be the main obstacles. A large number of the GP offices switched to teleconsultations to provide healthcare. Our monitoring instrument provided information for policy makers to intervene on a local level.

**Supplementary Information:**

The online version contains supplementary material available at 10.1186/s13690-022-01003-0.

## Background

In December 2019, the SARS-CoV-2 virus first emerged in Wuhan, China [1]. The COVID-19 outbreak has officially been characterized as a pandemic by the WHO since the 11^th^ of March 2020 [2]. On the 18^th^ of March 2020, the Belgian government declared a national lockdown to contain the spread of the virus [3].

Primary care faced an unprecedented situation, as it plays a central role in the healthcare response to the COVID-19 pandemic [4]. Work organisation in primary care has drastically changed worldwide. General practitioners (GPs) were among the first to confront the SARS-CoV-2 virus, which made them susceptible to infection. An explorative Australian study profiled the general practice response to the COVID-19 outbreak and showed a lack of personal protective equipment (PPE), a large increase in community anxiety and a high level of adaptability in GP offices (including switching to telephone consultations) [5]. An online questionnaire in France described how GPs adapted their practices to provide continuous healthcare to their patients, and it showed a major impact on a GP’s daily duties [6]. A survey in Shenzhen, a large city in China that was confronted with the SARS-CoV-2 virus in the early stages of the pandemic, showed that GPs had sufficient material and support, despite their clinical practice being afflicted by the virus outbreak [7]. A cross-sectional survey in the UK analysed how COVID-19 rapidly reformed primary care, with the most significant change being the enactment of teleconsultations and video consultations [8, 9]. Also in Italy COVID-19 pushed the shift towards digital care [10].

Additionally, in Belgium, the pandemic had a large impact on the way of working in general practice. Patients were encouraged to consult their GP by telephone, which protected them and their GPs. However, this measure also made it more difficult for GPs to assess their patients. Their practices suffered shortages in PPE and staff. Furthermore, Belgian GPs managed triage posts to relieve pressure on emergency rooms. Additionally, a substantial number of the GPs in Belgium combine their job with a coordinating function in a nursing home, some of which were greatly affected by the outbreak [9].

As GPs played a crucial role in the management of the COVID-19 pandemic and its consequences and because the pandemic strongly impacted their work, it was essential to monitor the feasibility of the GPs’ daily work during the first wave of the pandemic. Systematic monitoring of crucial indicators of GP centres might enable policy makers, on different geographical levels, to take timely measures to support GPs.

However, little is known about the burden primary care workers experienced during a critical period such as the COVID-19 pandemic. A weekly national American survey, conducted by the ‘Larry A. Green Center’, monitored and charted the impact on primary care [11]. In Belgium, a network of sentinel GPs, representative of the federated regions, is consulted on a regular basis on a wide range of topics, such as influenza outbreaks [12]. However, to gather daily information about the situation in GP centres, representative of a small geographical area such as an arrondissement, a tool is required that is both fast and causes as little additional burden for participating GPs as possible. To answer this need, we developed a monitoring instrument that allows semiautomated data collection from GPs. This paper describes the instrument that was installed and the results from monitoring the burden the COVID-19 pandemic caused on Belgian GPs during the first wave of the pandemic.

## Methods

### Design

We conducted a nationwide observational longitudinal prospective study. This study was approved by the Ethics Committee Research (EC Research) of the University Hospitals Leuven (UZ Leuven) (S63869) and the Belgian Information Security Committee, division social security and health (IVC/KSZG/20/218).

### Participants

Belgium is a federal state comprised of three regions: the Flemish region (Flanders) in the north, the Walloon region (Wallonia) in the south and the Brussels-Capital region. Flanders and Wallonia each represent five provinces. In total, Belgium has 43 administrative arrondissements. In Flanders, 60 primary care zones (approximately 100,000 inhabitants per zone) became operational during the first phase of the COVID-19 pandemic. Primary care zones have been established to better coordinate the work of local authorities, care providers and caregivers. Currently there is no counterpart in the French speaking part of Belgium.

On 31 December 2019, there were 16,722 active GPs in Belgium and 2,209 GPs in training [13]. Belgian GPs are organized into 153 different GP circles: 78 in Flanders [14], 61 in Wallonia [15] and 14 in Brussels. In total, 8 different electronic medical record (EMR) systems are used in general practice in Belgium.

### Measuring instrument

eForms (HealthConnect, Corilus, Gent, Belgium) allow a uniform and secure electronic exchange of structured data (e.g., FHIR, CSV, a custom defined format) between health care workers and health care organizations in Belgium and are integrated into EMR systems in general practice. eForms are typically used to exchange structured data on individual patients for a specific workflow. However, for the current study, an eForm to exchange aggregated data on the level of a GP centre was developed and made available for all Belgian GPs. The eForm was developed between the 17^th^ and 19^th^ of March 2020. At the beginning of the study, the eForms were not available in all EMR systems. Therefore, an alternative method for data collection using a web form was developed by Healthdata (www.healthdata.be), a federal platform organized by Sciensano (www.sciensano.be) to store health care data in Belgium. During the study, the eForms were implemented in all EMR systems.

The eForm and web form consisted of three parts: a part on the burden on the GP centre, an epidemiological part and a part on the availability of personal protection equipment (PPE). The first part contained five questions about the need for support, created by Vioras (Vioras, Tielt-Winge, Belgium, www.vioras.be): 1) were you able to perform your critical tasks today? 2) were you able to perform your tasks with a sufficient level of quality (according the guidelines)? 3) Do you need help? 4) Do you have enough resources (personnel and material) to execute your duties tomorrow? and 5) Do you have enough resources (personnel and material) to execute your duties next week? Questions were answered with yes or no, and extra information could be added in free text. The other questions concerned the presence and absence (due to an illness) of GPs in the GP centre and the workload in the GP centre (increased, normal or decreased). The epidemiological part questioned: 1) the number of telephone and physical consultations, 2) the percentage of consultations related to respiratory problems, and 3) the number of patients referred to a triage centre or the emergency department due to COVID-19 suspicion. The last part contained questions on the availability of PPE, namely, surgical masks, FFP2 masks, disinfectant hand gel, gloves and protective suits. The respondents indicated whether they had a significant shortage, a small shortage, a small surplus or a significant surplus for each category.

All Belgian GP centres had the opportunity to participate at any moment during the study period. GPs were informed by their EMR developer (email, website) that the eForm (and web form) was available and they were invited to participate. A member (GP) of each GP centre was asked to send an eForm or fill out the web form each day, reporting the situation at that centre. A reminder to fill out the eForm (or web form) was sent each day (except weekends and holidays) to all GPs that participated at least once. From the beginning of June 2020, GP centres were asked to send the eForm once a week instead of each day. Data from the 19^th^ of March until the 17^th^ of August 2020 were included.

The eForms were sent to the principal investigator of this project (BV), who is also a general practitioner, who integrated the received eForms automatically into a.csv file. This file was shared with Healthdata (www.healthdata.be). Healthdata integrated all data from the eForms and web forms and shared the data with Vioras (www.vioras.be), which created daily reports based on the five questions about the need for support.

### Reporting

Each day, a report was prepared for the chairmen of the GP circles with the situation in their circle (for example, see www.vioras.be). This report consisted of a graphical presentation showing all GP centres in their circle and a colour code representing the status of each centre (red: urgent action needed, orange: upcoming problems in the following day(s), light green: possible problems in the following week, dark green: everything under control, grey: no response). In addition to the graphical presentation, they also received a detailed report with all of the specific comments the GP centres registered. This report directed the actions of the circles to support the GP centres in their region.

Because the GP centres and circles were identified based on their geographical location, a hierarchical structure could be made, and overarching reports were also generated on the level of primary care zones (only in Flanders), arrondissements, provinces, federal regions and country to give policy makers tools to direct their actions.

### Data analysis

Descriptive statistics were used. The denominator to calculate the epidemiological incidences was based on the number of GPs per practice and the estimated number of patients per GP. This number was estimated separately for each district (arrondissement) in Belgium based on 2018 data on population statistics (www.statbel.fgov.be) and the number of active GPs, defined as GPs having $\ge$ 500 contacts/year (www.riziv.fgov.be). SAS 9.4 was used to perform the data analyses.

## Results

**Fig. 1:**
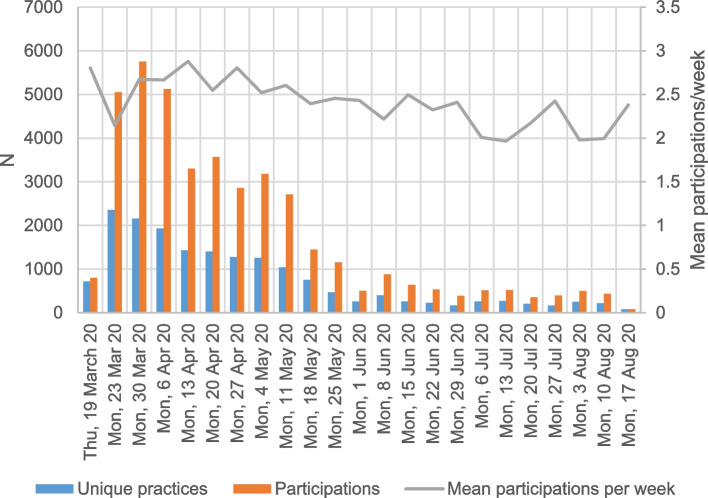
Number of participating practices and participations per week from March to August 2020 in Belgium

**Fig. 2:**
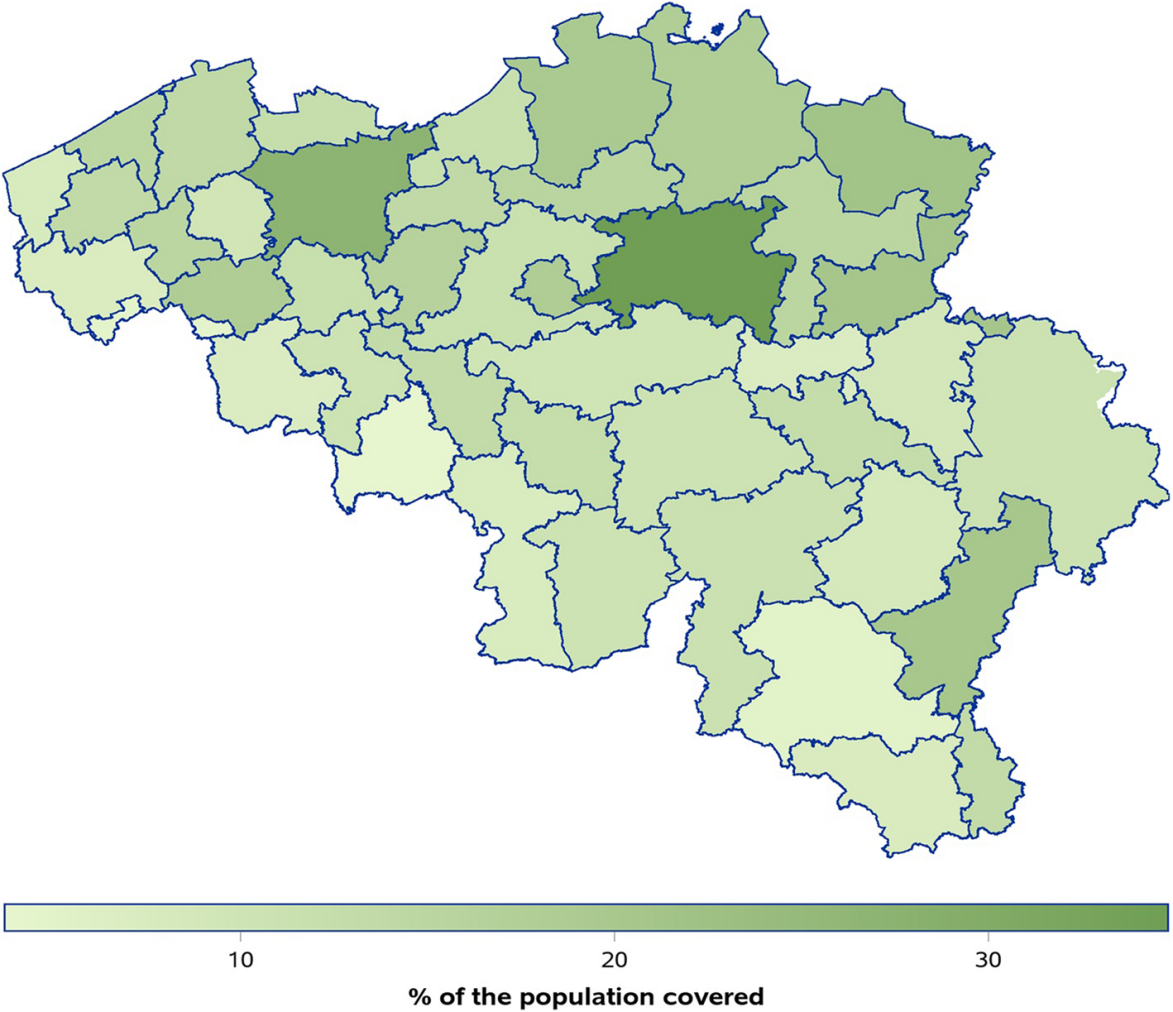
Coverage of population by participating practices per arrondissement in Belgium between March and August 2020

In total, 3,769 different GP centres participated at least once, which included more than 10,000 GPs (Fig. 1). Most often, eForms were used (> 90%) instead of the web form. During the study period the weekly number of participating GP centres decreased, but the participating centres continued to share their data on average at least twice per week. In certain arrondissements, the participating GP centres covered 25–30% of the total population during the first two months when participation was the highest (Fig. 2). The number of participating GP centres decreased progressively, as did the number of confirmed COVID-19 cases (confirmed cases in Belgium, see https://epistat.wiv-isp.be/covid/covid-19.html).

### Burdens on the GP centres

Throughout the first 3 weeks, at least 20% of participating GP centres declared they had insufficient resources for the following week in terms of equipment and staff. Approximately 10% continued to report this throughout the entire study. The number of GP centres indicating not ready for the next day remained at approximately or under 10% at any given time. Between 98% and 99.75% reported that they were able to complete their daily essential tasks. In those cases, more than 94% were able to execute these tasks meeting the expected standards in terms of quality of care.

During the first weeks of the pandemic, the absence of GPs varied between 3 and 6%. Afterwards, the percentage remained below 3%. Since the beginning of July, none of the practices reported any absentees due to illness.

**Fig. 3:**
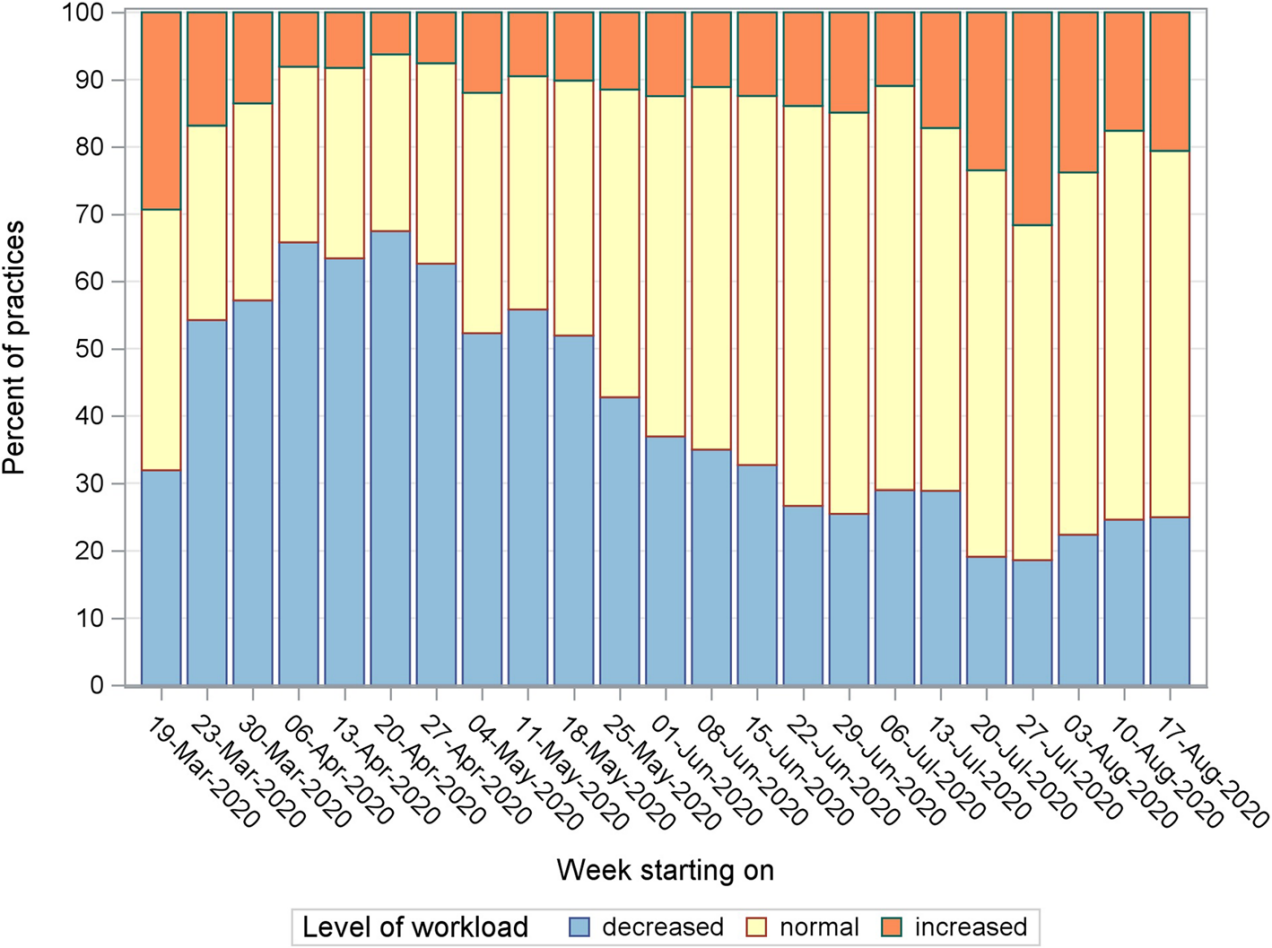
Level of workload in general practice from March to August 2020 in Belgium

During the first week, 30% of the GP centres indicated an increased workload. From the second week onwards, this number decreased and stabilized at an average of 10–20%. We noticed another rise in workload over the course of July and August. A sizable number of GPs indicated a decreased workload, especially during the first half of the monitoring period (Fig. 3). The percentage of GP centres that endured an increased workload was comparable across the three Belgian regions (Table 1).

**Table 1:** Heterogeneity across the three Belgian regions in March, April and June 2020

	**Flanders**	**Wallonia**	**Brussels**
**Week of 19 Mar 2020**	*N* = 562	*N* = 188	*N* = 55
Increased workload	30%	28%	31%
Large shortage of surgical masks	17%	29%	16%
Large shortage of FFP2 masks	54%	72%	67%
**Week of 6 Apr 2020**	*N* = 3290	*N* = 1329	*N* = 491
Increased workload	9%	6%	9%
Large shortage of surgical masks	19%	17%	20%
Large shortage of FFP2 masks	26%	49%	59%
**Week of 8 Jun 2020**	*N* = 658	*N* = 144	*N* = 81
Increased workload	12%	6%	9%
Large shortage of surgical masks	1%	3%	1%
Large shortage of FFP2 masks	20%	26%	23%

### Epidemiology

**Fig. 4:**
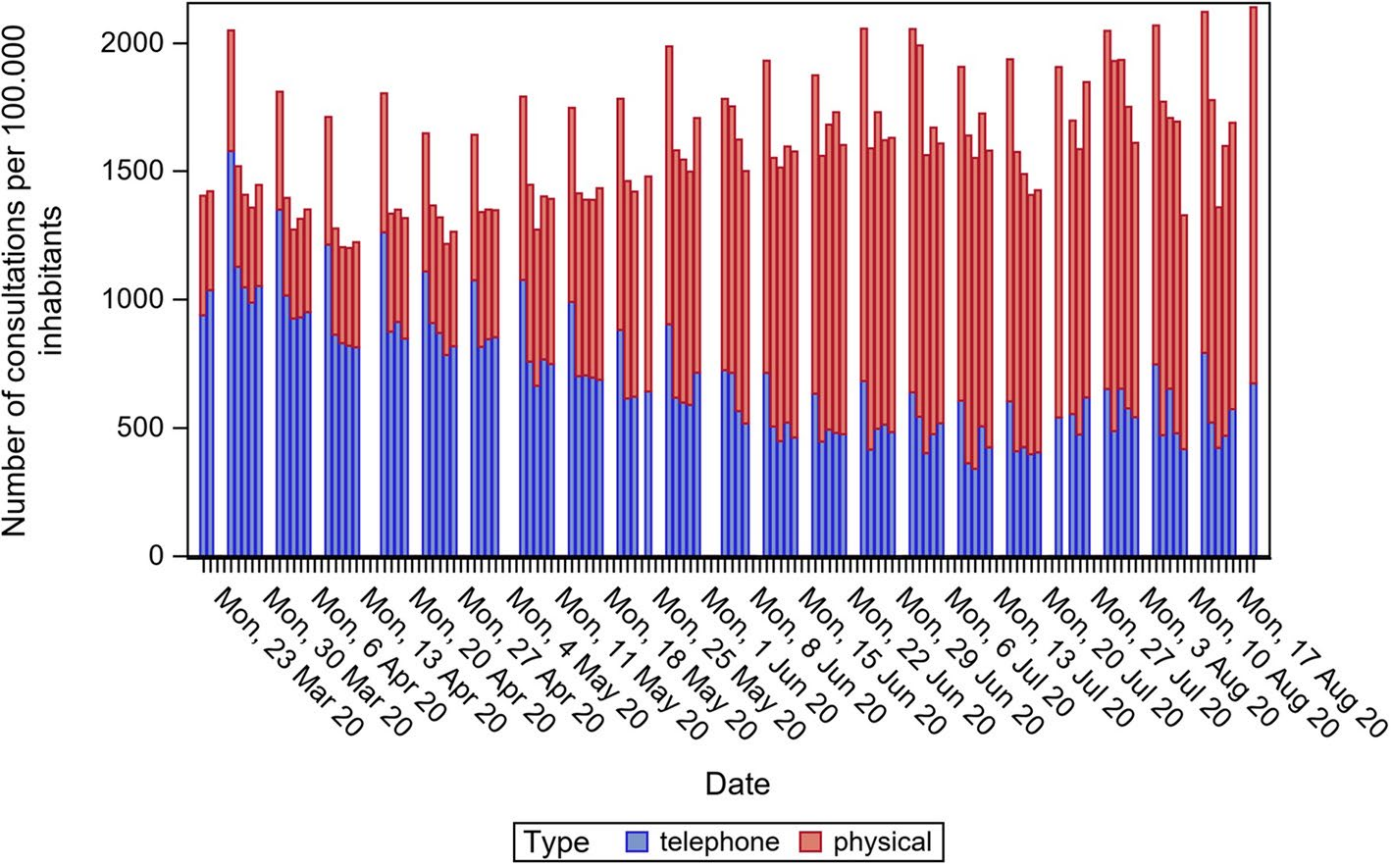
Number of consultations per 100,000 inhabitants in general practice from March to August 2020 in Belgium

**Fig. 5:**
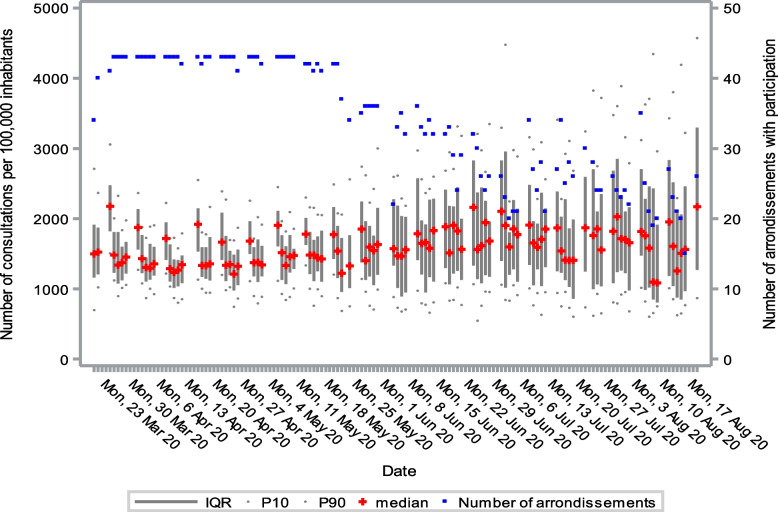
Variability of number of consultations in general practice per arrondissement from March to August 2020 in Belgium

**Fig. 6:**
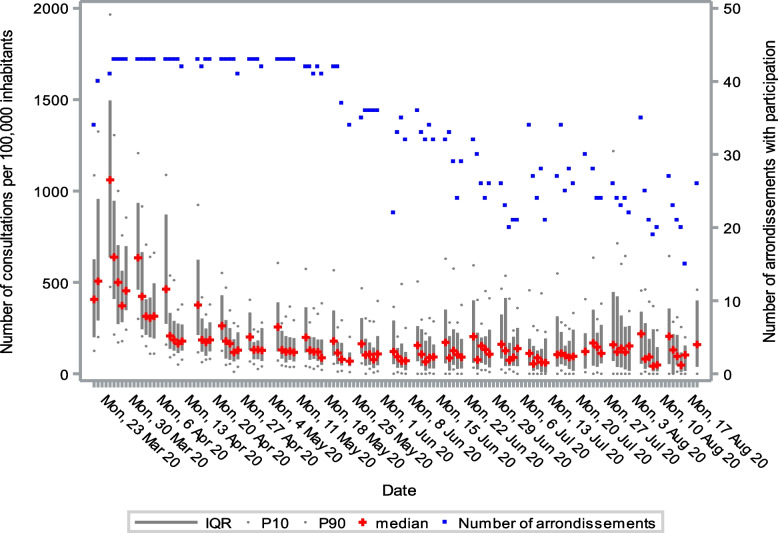
Number of consultations in general practice related to respiratory problems from March to August 2020 in Belgium

The number of consultations per 100,000 inhabitants remained steady throughout the entire monitoring period. More than 70% of the consultations in March were conducted by telephone. This percentage started to decline in April and May and stabilized in June at approximately 30% (Fig. 4). Figure 5 shows the variability in number of consultations per arrondissement. The number of consultations due to respiratory symptoms decreased considerably over the first few weeks of the study. From there on, the numbers stabilized until the beginning of August, where we observed a limited increase (Fig. 6). In general, Monday was the most busy day of the week for GP centres.

Most of the referrals to the emergency department were reported during the first weeks of the monitoring period. From week 7 (27^th^ of April to the 3^rd^ of May) to week 8 (4^th^ to the 10^th^ of May), the number of referrals to the triage centres more than doubled and featured an increasing trend from that point onwards (data not shown).

### Personal protective equipment

**Fig. 7:**
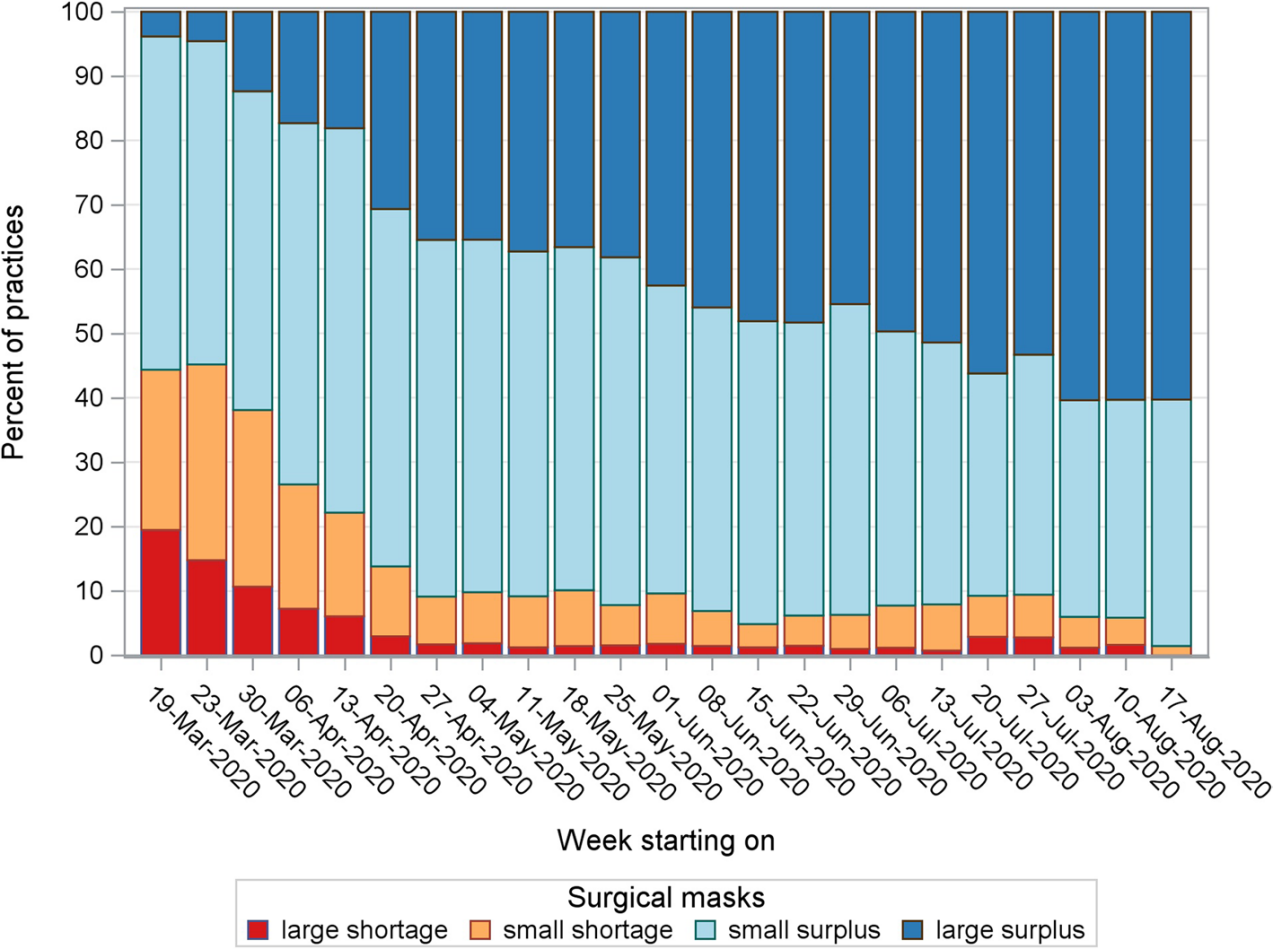
Shortage of surgical masks in general practice from March to August 2020 in Belgium

**Fig. 8:**
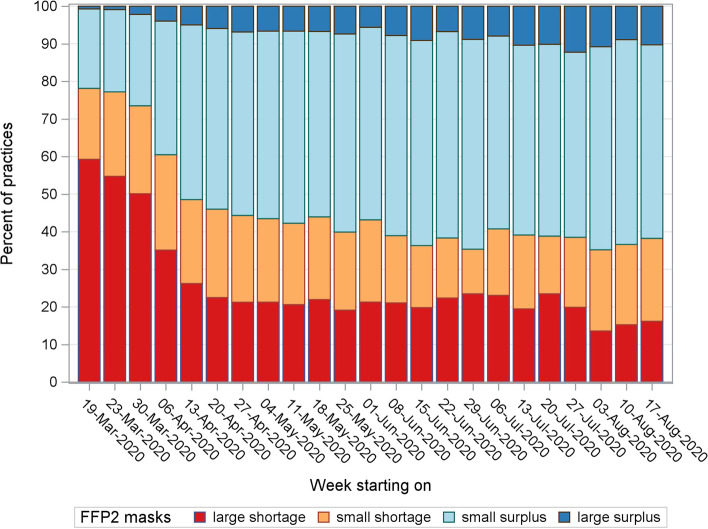
Shortage of FFP2 masks in general practice from March to August 2020 in Belgium

During the first 2 weeks of the survey, 40% of the GP centres reported having at least a minor shortage of surgical masks and disinfectant hand gel. From the beginning of May onwards, the deficit in surgical masks decreased and stabilized at a maximum of 10% (Fig. 7). The hand gel followed this trend but stabilized at a maximum of 20% (data not shown). At least 70% reported having a lack of FFP2 masks during the first 2 weeks (Fig. 8). A large shortage was less pronounced in Flanders compared to the other two regions (Table 1). During the remainder of the survey, the scarcity of these masks persisted in approximately 40% of the GP centres.

### Reporting

The Flemish GP circles in Flanders and Brussels received their daily report from Vioras by email from Domus Medica (www.domusmedica.be). Domus Medica represents the interests of GPs and GP circles in Flanders on scientific, social and syndical levels. Between the 4^th^ of May and the 14^th^ of May, all 78 chairmen of the Flemish GP circles were contacted. In total, 74 chairmen were interviewed, of whom 34 (46%) reported having undertaken action based on the daily report. These actions were mainly directed towards solving the shortages of PPE. In Wallonia and the French speaking part of Brussels, no representation of the GP circles was found to diffuse the daily report to the GP circles. The coordinators of the 60 primary care zones in Flanders each week received a report on the situation in the GP centres in their primary care zone from Vioras through VIVEL (www.vivel.be). VIVEL is the Flemish Institute for Primary Care, which has been financed by the Flemish Government as a partner organization for primary care since 1 May 2019. At the Flemish level, VIVEL is the central point of contact and the platform for the dialogue between the first-line actors with the government and with each other. From June onwards, the daily and weekly reports by Vioras were no longer diffused. All collected data were made available to the governments at different administrative levels (regions and country) by the Healthdata platform.

## Discussion

This study introduced an instrument to monitor the need for support and the workload, the epidemiology and the need for personal protection equipment in Belgian GP centres. A large number of GP centres participated mainly during the first 3 weeks of the first wave of the COVID-19 outbreak. A stunning 20% of the GP centres reported insufficient resources to take on the upcoming week. Importantly, despite this situation, the large majority of general practices reported being able to complete their daily tasks and provide quality care to their patients. However, it should be mentioned that chronic care was largely put on hold during the first wave in Belgium.

We noticed that a substantial proportion of the GP centres had an increased workload, which gradually decreased over time along with the intensity of the epidemic. The gain in workload reported in July was presumably a result of GPs leaving for vacation and changed COVID-19 testing criteria, which increased the number of people who needed a test, for example, after high-risk contact or after returning from vacation.

Furthermore, we observed a drastic change in the way patients consulted their GPs. A major portion of the consultations (> 70%) were conducted telephonically, especially during the first half of the reported period. In France, 91.7% of the GPs declared an increase in teleconsultations [6]. In Australia, 97% reported a high or medium use of video and/or telephone consultations since the availability of telehealth reimbursement [5] Teleconsultations already seemed to be commonly used in the UK, yet most of the GPs reported an increase. Most fascinating was the increase in the usage of video consultations, rising from 3 to 95% of the responders [8]

The number of consultations due to respiratory symptoms dropped considerably over the first weeks, probably due to the strict lockdown measures that severely limited the amount of contact among people (see Additional Figure). Referrals to the emergency department peaked at the beginning of the outbreak. Later, referrals to triage centres started to rise. The increase in the number of referrals to triage centres at the beginning of May was a result of the government invoking new criteria for testing symptomatic patients for COVID-19. In July, these criteria changed again, allowing asymptomatic patients to be tested as well. For example, people who had been exposed to COVID-19-positive cases or were returning from abroad.

There seemed to be a large number of GP centres that suffered a shortage of PPE throughout the first weeks. The lack of surgical masks and disinfectant hand gel partially diminished over time. However, the shortage of FFP2 masks remained problematic.

At the beginning of the COVID-19 pandemic, GP centres and policy makers were overwhelmed with information and fast-changing guidelines. This led to much stress and changing working methods in the field. An American survey conducted by the ‘Larry A. Green Center’ monitored US primary care on a national level since the beginning of March [11]. The survey aimed to provide an overview of the burden on primary care to support policy makers in crisis decision making. In contrast to our project, the results were posted after every survey separately, without the context of a longitudinal view. Furthermore, the data captured by our monitoring instrument were visualized and structured in daily and weekly reports for chairs of GP circles and policy makers at different levels. However, since this introduced a completely new approach to guiding policy and because of the complex Belgian federal structure, we were confronted with multiple barriers in trying to implement these reports. This led to only a partial uptake of the collected data and visualizations to guide policy.

### Strengths and limitations

By using the eForms, we were able to collect reliable data consistently and daily. During the study, the eForms were incorporated into all Belgian EMR systems available in general practice. It only took a couple of minutes to fill out the eForm, and the GPs received a daily reminder. By starting early in the outbreak, our study captured almost the entire first wave in Belgium. This instrument enabled a quick follow-up on the need for support and shortages of PPE in Belgian GP centres to inform policy makers and guide their decisions.

However, a few limitations should be noted. First, this project was possibly exposed to selection bias, excluding GPs that were less IT minded. Moreover, GP centres that endured great pressure or encountered many COVID-19-related cases may have not been prone to send the eForm because of a lack of time. This might have led to an underestimation of the reported workload. However, when a GP centre stopped sending eForms and turned ‘grey’ in the reports to policy makers, this should be seen as a signal that the GP centre is possibly facing problems and is not able to answer. On the other hand, the high number of infections and sick GPs during the first 3 weeks of the pandemic also might have inflated negative responses and thus increasing the percentage of GP centres that reported insufficient resources to take on the upcoming week. Second, absentees of staff due to illness in GP centres seemed to be limited and generally only present during the onset of the outbreak. However, these were not specified to be due to COVID-19, and they may have also been absent for other reasons. Furthermore, we suspect this number to be underestimated, as solo practices may have not been able to respond to the survey. Third, the percentage of consultations due to respiratory complaints was a very rudimentary epidemiological measurement. This was used because of the vague case definition of COVID-19 at the beginning of the outbreak. On the other hand, most information was captured by the longitudinal nature of this measurement. Fourth, the lack of PPE that persisted over the last weeks of the survey may be overestimated. Reaching the closure of the survey, the number of participating GP centres declined. Possibly, the remaining practices were those who were still confronted with a shortage of PPE. Last, we did not collect data on the severity of cases treated by the GPs. Therefore, we were not able to investigate whether the workload was associated with the severity of the cases instead of the volume of the visits. Furthermore, preventive care and diagnostic tests were probably forgone by many patients during the pandemic. In this perspective a recent publication with data from the Flemish Intego morbidity registry showed that during the first COVID-19 year, overall care provision and the incidence of acute diagnoses increased, whereas chronic diseases' incidence decreased. More granular, care provision and chronic diseases' incidence decreased during the lockdowns, especially for people with a lower socio-economic status. After the lockdowns they both returned to baseline [16].

### Implications

By implementing these results in a demographic overview (www.vioras.be), we gained more detailed insight into where GPs were in need of support and where to intervene. In other words, these data could help policy makers guide their actions and support their GP centres on a local level, for example, in the redistribution of personal protection equipment, based on the principle of solidarity. Therefore, this technology introduces many possibilities for guiding future projects in general practice. However, it will be important to identify and train policy-makers at different policy levels to work with the reports, possibly by introducing small-scale projects that can be gradually expanded.

Although we monitored only the number of consultations due to respiratory symptoms, this rudimentary indicator might be used as an indicator for predicting the number of COVID-19 hospitalizations and intensive care admissions in the following 2 weeks. A Belgian study showed that the diversity in the number of COVID-19 cases could be partially explained by model-based symptom incidence predictions [3]. Therefore, based on these data, we might identify a window of opportunity to undertake actions in a timely manner to avoid an increase in hospitalizations.

## Conclusion

We introduced an instrument in Belgian EMR systems to monitor the burden on GPs during the first wave of the COVID-19 pandemic. The lack of PPE and the increase in workload were considered to be the main obstacles. A large proportion of the GP offices switched to teleconsultations to provide healthcare. Our monitoring instrument provided information for policy makers to intervene on a local level.

## Supplementary Information

**Additional file 1. **Study timeline in relation to the manifestation of the pandemic in Belgium from March to August 2020 in Belgium. Source: https://epistat.sciensano.be/Covid/#Data

## Data Availability

The datasets used and/or analysed during the current study are available from the corresponding author on reasonable request.
