# Supplementary figures and images for: Burden of COVID-19 on primary care in Belgium: a prospective nationwide observational study from March to August 2020
Source: Arch Public Health. 2022 Dec 8;80:250. doi: 10.1186/s13690-022-01003-0 (PMC9730669; doi:10.1186/s13690-022-01003-0)

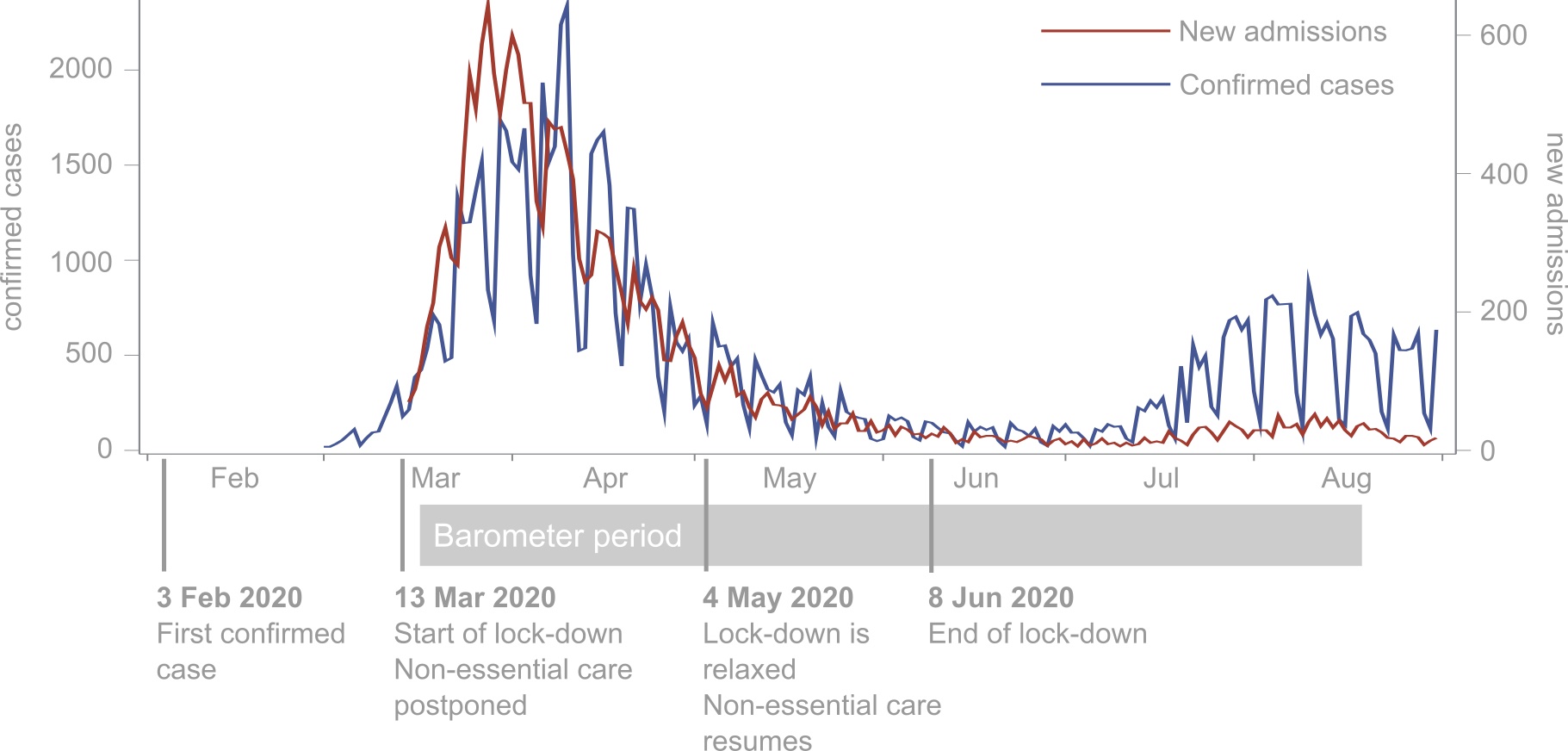

Supplement: Supplementary file 1 — Additional file 1. Study timeline in relation to the manifestation of the pandemic in Belgium from March to August 2020 in Belgium. Source: https://epistat.sciensano.be/Covid/#Data [file 13690_2022_1003_MOESM1_ESM.jpg]
